# Comparative evaluation of group-based mindfulness-based stress reduction and cognitive behavioral therapy for the treatment and management of chronic pain disorders: protocol for a systematic review and meta-analysis with indirect comparisons

**DOI:** 10.1186/2046-4053-3-134

**Published:** 2014-11-10

**Authors:** Taylor Hatchard, Chris Lepage, Brian Hutton, Becky Skidmore, Patricia A Poulin

**Affiliations:** 1 School of Psychology, University of Ottawa, Ottawa, Canada; 2 Ottawa Hospital Research Institute, Ottawa, Canada; 3 Faculty of Epidemiology and Community Medicine, University of Ottawa, Ottawa, Canada; 4 Department of Psychology, The Ottawa Hospital, Ottawa, Canada; 5 Department of Anesthesiology, The Ottawa Hospital, Ottawa, Canada

**Keywords:** Mindfulness-based stress reduction, Cognitive behavioral therapy, Chronic pain, Meta-analysis, Systematic review, Randomized controlled trials

## Abstract

**Background:**

Chronic pain disorders impact the physical, psychological, social, and financial well-being of between 10%–30% of Canadians. The primary aims of psychological interventions targeting chronic pain disorders are to reduce patients’ pain-related disability and to improve their quality of life. Cognitive behavioral therapy (CBT) is the prevailing treatment for chronic pain, however mindfulness-based stress reduction (MBSR) has displayed promise as an alternative treatment option. The objective of this systematic review and meta-analysis is to compare MBSR to CBT in their relative ability to reduce pain-related disability and intensity, to alleviate emotional distress, and to improve global functioning in chronic pain patients.

**Methods/design:**

We will conduct a systematic review with meta-analyses to compare MBSR to CBT in the treatment of chronic pain disorders in adults. We will report our review according to the recommendations provided by the PRISMA statement. Randomized studies will be included and the literature search will comprise Ovid MEDLINE®, Ovid MEDLINE® In-Process & Other Non-Indexed Citations, Embase Classic + Embase, PsycINFO, the Cochrane Library on Wiley, including CENTRAL, Cochrane Database of Systematic Reviews, DARE, and HTA. Study selection and data extraction will be conducted by independent investigators and in duplicate. Outcomes of interest will include pain interference, pain intensity, emotional functioning, and patient global impression of change. The Cochrane risk of bias tool will be used to assess risk of bias of included studies. As we anticipate that scales used to measure participant responses will be related but varied from study to study, standardized mean differences will be used to compare effect sizes between treatment modalities. Given the possibility of little or no head-to-head evidence comparing MBSR with CBT, we will use indirect treatment comparison methodology to assess the relative effectiveness of these interventions.

**Discussion:**

The findings from this study will assist patients and treatment providers to make informed decisions regarding evidence-based treatment selection for chronic pain disorders.

**Systematic review registration:**

PROSPERO CRD42014009356

## Background

Chronic pain disorders are multidimensional, often characterized by physical, psychological, social, and financial suffering and affect as many as 10%–30% of Canadians [1]. Chronic pain disorders often result in high rates of depression and insomnia, as well as increased rates of stress, anxiety, and other emotional problems [2,3]. The purpose of many psychological interventions aimed at alleviating the impact of chronic pain disorders is primarily to assist patients in developing autonomy in coping with their condition, regain their sense of purpose, recover their strength, reduce pain-related disability, and ultimately improve their quality of life [4].

Although a plethora of treatments are available for chronic pain disorders, cognitive behavioral therapy (CBT) is currently the dominant psychological intervention for such conditions [5]. The aim of CBT is to help patients learn how to think and behave in more adaptive ways. In the context of chronic pain, CBT components often include cognitive restructuring of maladaptive pain-related beliefs, coping skills training, problem-solving training, and psychoeducation of pain and their particular syndrome. CBT also often includes several behavioral strategies as well, such as relaxation training, strategies for behavioral activation, pacing, activity scheduling, and motivating physical activity [4].

Another promising intervention is mindfulness-based stress reduction (MBSR), which was originally developed by Kabat-Zinn [6] to treat and manage chronic disorders. MBSR is a group-based intervention that focuses on improving awareness and acceptance of moment-to-moment experiences, including physical discomfort and difficult emotions. The core of MBSR consists of mindfulness exercises that serve to increase awareness of sensations, emotions and thoughts, to provide self-regulation strategies, and to promote healthy and adaptive responses to stress.

The standard MBSR program requires one 2 to 2.5-h session per week for a duration of 8 weeks, as well as a 1-day session of intensive practice. Program components include different mindfulness meditation exercises, with different foci (e.g., body sensations, breath, thoughts). Each class has a didactic component and group discussions. In between sessions, participants are assigned up to 45 min of daily practice of the MBSR components at home which is generally supported by audio recordings and handouts. Furthermore, participants are encouraged to integrate mindfulness into their daily activities through choosing routine activities (e.g., showering, washing dishes) and executing these activities in a mindful way through focusing fully on the experience of the task at hand.

One of the ways that mindfulness is purported to be efficacious in the treatment of chronic pain disorders is through the development of equanimity in the presence of unpleasant experiences and the ability to respond instead of automatically reacting to challenges, including pain [6]. Being responsive instead of reactive towards stressors can lead to the adoption of more adaptive coping strategies. Furthermore, the practice of mindfulness can improve acceptance through facilitating the grieving of the unavoidable losses that accompany life with chronic pain, which in turn is associated with better overall outcomes [7]. Finally, the practice of mindfulness meditation is also associated with neuroendocrine and immunological changes that may also mediate some of the cognitive benefits reported by participants [8].

### Purpose of the proposed meta-analysis

Since the original study in 1982 [5], a number of subsequent investigations have been conducted that evaluated the efficacy of MBSR training for chronic pain conditions [9]. The purpose of the proposed systematic review and meta-analysis is to quantify the efficacy of MBSR within this population and to directly compare it to the efficacy of group-based CBT interventions, the current most commonly used treatment. This will establish whether MBSR differs in effectiveness from group-based CBT for outcomes of interest related to pain symptoms.

We will assess treatment efficacy based on criteria outlined in the interpreting the clinical importance of treatment outcomes in chronic pain clinical trials (IMMPACT) recommendations [10]. Our primary outcome of interest will be pain interference (i.e., reduced disability), and our secondary outcomes will be pain intensity, emotional functioning, and global rating of improvement. We hypothesize that MBSR and CBT will not differ in terms of treatment benefits for both our primary and secondary outcome measures of interests. We are not aware of any existing studies comparing CBT and MBSR directly, and thus, evidence synthesis methods enabling indirect comparisons between interventions are likely to be helpful.

## Methods/design

### Study design

The proposed systematic review and meta-analysis will be conducted in accordance with the reporting guidance included in the preferred reporting items for systematic reviews and meta-analyses (PRISMA) statement.

### Study registration

This meta-analysis is registered with PROSPERO (CRD42014009356).

### Study eligibility criteria

#### ***Type of studies***

We will include randomized controlled trials that have evaluated the efficacy of MBSR or CBT programs for any chronic pain disorder. This will include treatment groups compared with standard care, treatment groups compared with wait-list/no-treatment conditions, and treatment groups with adjunctive treatments compared with the same adjunctive treatments alone.

#### ***Type of participants***

We will include studies of all adults (i.e., ≥18 years old) with chronic pain conditions in both treatment and control participants. We will adopt the definition of pain provided by the International Association for the Study of Pain, which states that pain is an ‘unpleasant sensory and emotional experience, associated with actual or potential tissue damage, or described in terms of such damage’ [11]. To be considered chronic, the pain must have been present or recurrent with a minimum of 3 months duration at the time of intervention. Chronic pain conditions include rheumatoid arthritis, arthralgia, temporomandibular joint syndrome, myofascial pain condition, neck pain, back pain, neuralgia, myalgia, myodynia, chronic compartment syndrome, rheumatic polymyalgia, and fibromyalgia, with the exception of migraines and headaches due to the different emphasis of treatment in these conditions compared to other chronic pain disorders. Studies that enrolled children or patients who had been experiencing pain for less than the 3-month threshold duration will be excluded from the present study.

#### ***Type of interventions***

Eligible MBSR programs must adhere to the standardized program format developed by Kabat-Zinn [6]. The program is offered in group format and typically requires 8 to 10 weekly, 2 to 2.5-h sessions, as well as a 1-day session of intensive practice and 45 min of daily home practice. We will accept studies of the program with relatively minor deviations and document them. Studies using other mindfulness-based programs, such as mindfulness cognitive therapy, will be excluded from our analyses, as we are only interested in the MBSR program for the purposes of this investigation. Eligible CBT programs must be delivered in group, in-person formats. Duration of CBT programs is typically more variable than MBSR, and as such, we will include all programs regardless of length. Programs should include specific techniques often used for pain treatment and management, including relaxation training, cognitive restructuring (i.e., changing pain-related beliefs, reducing rumination, etc.), setting and working towards behavioral goals (e.g., exercise), behavioral activation, and problem-solving training. Adjunctive treatments of relevance, which may be given in combination with these therapy programs, will include medical interventions such as pharmaceutical treatment. As noted earlier, eligible interventions will also include standard care groups and wait-list/no-treatment conditions given the anticipated need for indirect comparison methods to compare MBSR with CBT.

#### ***Type of outcome measures***

We are primarily interested in outcomes that measure change in pain interference from pre to post MBSR or CBT treatment as an index of improvement in patients’ physical functioning. Secondary outcomes of interest include pain intensity, emotional functioning, and patients’ global impression of change. These variables are commonly measured using psychometric tools with demonstrated reliability and validity. This includes the Brief Pain Inventory (BPI; [12]) and Multidimensional Pain Inventory (MPI; [13]) to measure pain interference, the Beck Depression Inventory (BDI; [14]) and Profile of Mood States (POMS; [15]) to measure emotional functioning, and the Patient Global Impression of Change (PGIC; [16]) to measure global rating of improvements. Lastly, changes in pain intensity are generally measured through a numerical rating scale from 0–10 [10].

### Search methods for identification of studies

Electronic search strategies were developed and will be tested through an iterative process by an experienced medical information specialist in consultation with the review team. Using the OVID platform, searches will be performed on Ovid MEDLINE®, Ovid MEDLINE® In-Process & Other Non-Indexed Citations, Embase Classic + Embase, and PsycINFO. We will also search the Cochrane Library on Wiley (including CENTRAL, Cochrane Database of Systematic Reviews, DARE, and HTA). A grey literature search will also be carried out in using CADTH’s *Grey matters: a practical search tool for evidence-based medicine *[17].

Strategies will utilize a combination of controlled vocabulary (e.g., pain; mindfulness; cognitive therapy) and keywords (e.g., myalgia, meditation, CBT). Vocabulary and syntax were adjusted across databases. There will be no date or language restrictions used. Additional references will also be sought through hand-searching the bibliographies of the included studies. Specific details regarding the full search strategies are provided in Additional file 1.

### Selection of studies

Study selection will be conducted in duplicate by eight independent evaluators that will be paired into teams in order to screen articles. Prior to screening, evaluators will be trained on the purpose of the study, the treatments being investigated, and specific criteria for inclusion and exclusion. This will be done didactically and through pilot screening on a sample of abstracts. This will be followed by the screening of study titles and abstracts for potential inclusion (i.e., stage 1 screening). Studies that are identified as potentially relevant during stage 1 will then undergo full-text screening by the four of the eight evaluators (stage 2 screening). Disagreements among the evaluators regarding study eligibility following abstract and full-text review will be resolved through consensus.

### Data extraction

Two independent evaluators will be responsible for collecting primary data from the included trials, which will be stored in Microsoft Excel. We will extract data relevant to our primary and secondary outcomes, which will include the mean (M), standard deviation (SD) (or standard error of the mean (SEM)), and the sample size (*N*) for both the treatment and control groups for each continuous outcome at baseline and post-treatment (as well as the difference of within-group changes and the corresponding measure of variation when reported). We will also collect information related to patient characteristics, such as age, chronic pain disorder, location of pain, average time since diagnosis, work status, pain medication, co-morbid mental health, as well as characteristics related to the intervention, such as treatment adherence, therapist competence (e.g., type/presence of professional training, years of experience), and information about home practice adherence. Discrepancies between the two evaluators in extracted data will be resolved through review by a third evaluator.

### Risk of bias assessment

The methodological quality of included randomized trials will be measured through a risk of bias assessment conducted by two independent evaluators using the Cochrane Collaboration’s tool for assessing risk of bias [18]. The assessment tool includes items related to randomization, allocation concealment, blinding of participants and outcome assessment, incomplete outcome data, selective reporting, among others. Studies will also be evaluated based on a number of important factors considered by the authors to be potentially related to intervention quality. As noted above, this includes recording studies’ measures of treatment adherence, therapist competence (e.g., type/presence of professional training, years of experience), and information about home practice adherence (see Table 1 for coding description).

**Table 1:** Description of additional intervention items to be rated during the risk of bias assessment

**Item**	**Points**	**Description**	**Examples**
Treatment adherence	1	Adherence to treatment protocol was measured by an independent evaluator.	
	0	Measurement of treatment fidelity is inappropriate or not mentioned.	If adherence was measured but was not conducted by an independent evaluator, a score of 0 will be assigned.
Therapist competence	1	Mention of therapists’ formal training in MBSR or CBT.	
	0	Method of therapist competence is inappropriate or not mentioned.	If formal training was omitted, a score of 0 will be assigned.
Home practice adherence	1	Home practice adherence was compared with outcome measures.	
	0	Method of home practice adherence is inappropriate or not mentioned.	If home practice adherence was not compared with outcome measures, a score of 0 will be assigned.

Treatment adherence refers to quantifying the degree to which an intervention has been delivered in accordance with its intended format. Measuring treatment adherence is critical in psychosocial interventions in order to ascertain whether treatment studies have truly manipulated the independent variable of interest (i.e., treatment) and if observed outcomes are truly based on the treatment itself or whether results have been influenced by confounding factors [19]. Similarly, it is also important to evaluate the competence of the therapist delivering the interventions, through both formal training and years of experience in delivering the treatment. This will ensure that the described treatment was conducted in a valid and reliable manner in order to draw firm conclusions regarding the effectiveness of the treatment in question [20]. Lastly, compliance with home practice requirements is an important factor in developing gains from program participation; although findings have been variable, a significant relationship between the time engaged in home practice and greater symptom improvement has been demonstrated in a number of studies [21-23]. This information collected on therapist competence, treatment adherence, and home practice adherence will be used to describe variations in treatment studies and will be used to highlight important gaps in the current body of literature.

### Analysis

Standardized mean differences (SMD) will be computed to obtain a summary measure of effect size across studies in order to quantify the impact of treatment relative to controls, as this will allow us to synthesize data measuring the same outcomes (e.g., change in pain interference) when used different scales from each other to measure these outcomes. As we anticipate little to no evidence directly comparing the benefits of MBSR relative to those associated with CBT within single studies, network meta-analysis [24,25] as described further below will be used to explore comparisons of these therapies. Recent research suggests that different types of control therapy may be associated with different effect sizes for CBT (and likely other forms of psychotherapy as well [26]). Furthermore, we will explore the impact of adjunctive medications in addition to these therapy programs by reflecting them distinctly in treatment networks relative to interventions of the therapy programs offered without these medications. We will avoid lumping of different types of control groups in primary analyses. If these analyses demonstrate similar benefits of MBSR and CBT relative to different control interventions, lumping of control groups will be considered in order to work with a more parsimonious model. An analogous strategy involving the use of adjunctive medical therapies will be considered.

Content experts will review tables summarizing key study characteristics to assess clinical and methodological heterogeneity of the evidence base. Prior to performing network meta-analyses, we will perform pairwise meta-analyses for each pair of treatments with available evidence to assess for the presence of statistical heterogeneity using Cochran’s *Q* (*p* value <0.10) and the *I*^2^ measure statistic (*I*^2^ > 50%). If our review of study characteristics or measures of statistical heterogeneity identify potentially important heterogeneity between studies, we will use subgroup and/or meta-regression analyses as appropriate to explore and account for key effect modifiers; this will include average patient age, type of chronic pain disorder, length of pain duration (i.e., as measured by average disease duration within each study), study level risk of bias (including therapist competence, treatment adherence, and home practice adherence), and so forth. We will accordingly account for these factors in indirect treatment comparisons. Additionally, key characteristics related to the patient population (i.e., age, chronic pain disorder, length of pain duration, etc.), interventions, and primary and secondary outcomes, and risk of bias assessment of included studies will be narratively summarized.

All pairwise meta-analyses will be performed using Comprehensive Meta-Analysis 2.0 software (CMA; Biostat, Englewood NJ, USA). WinBUGS software (MRC Biostatistics Unit, UK) will be used to perform network meta-analyses based on established methods [24,27,28]. This will include fitting of fixed and random effects meta-analyses including correlation adjustments for multi-arm trials as described elsewhere. We will compare each model’s residual deviance to the total number of data points (i.e., intervention arms) in the analysis to ensure these are approximately equal, suggesting adequate model fit. We will compare the deviance information criterion (DIC) obtained from fixed and random effects models to select between fixed and random effects models, with a difference of 5 or more points considered indicative of an important difference. Gelman-Rubin and trace plots will be reviewed to ensure convergence of the models. To explore the effects of potential sources of heterogeneity, network meta-regressions considering the covariates described above will be considered [24].

## Discussion

Despite its acceptance as the ‘gold standard’ in the treatment of chronic pain, a sizeable proportion of chronic pain patients do not respond positively to CBT [5]. Although a number of recommendations have been proposed to improve CBT for chronic pain patients [29], an additional solution may be to offer patients a different treatment option. MBSR was originally developed to manage and treat chronic pain disorders and it has shown a promise in its ability to improve pain severity and reduce psychological distress [30] in patients with chronic pain. Presently, it remains unclear how MBSR compares to CBT in the treatment of chronic pain disorders. We have planned this review to address this current knowledge gap. Based on anticipation of little to no information directly comparing the benefits of MBSR and CBT for this indication, we plan to use indirect comparison methods to derive estimates of their relative benefits. Such approaches to comparing treatments are increasing rapidly [31] and are helpful for decision-making.

### Limitations

The applicability and generalizability of the findings from the current study will have certain limitations. First, although treatment providers often modify the MBSR program, we will only include studies that have employed its standardized version, with minor deviations. Similarly, only studies that have incorporated group-based CBT will be included in our analyses. Finally, we will only include studies of interventions in chronic pain in patients aged 18 years or older, limiting our findings to the adult population.

## Abbreviations

CBT: cognitive behavioral therapy; MBSR: mindfulness-based stress reduction.

## Competing interests

The authors declare that they have no competing interests.

## Authors’ contributions

TH led the project and was responsible for the included conceptualization of the project, literature review, manuscript writing, and consultation with other team members. CL was primarily responsible for writing certain sections of the manuscript, researching aspects related to protocol design, general formatting, and assisted with conceptualization of the project. BH was primarily responsible for conceptualizing aspects of the analysis, writing, and providing feedback/editing of the manuscript. BS was primarily responsible for the literature search strategy. PP was the supervising researcher on this project, who was primarily responsible for assisting with the conceptualization (e.g., what disorders to include), providing support and guidance with methodology, writing, and editing the manuscript. All authors read and approved the final manuscript.

## Supplementary Material

Additional file 1 **Search strategy.** Literature search strategy contains keywords and search strategies used to obtain articles for screening and data extraction for the meta-analysis. Click here for file
