# Supplementary material for: Comparative evaluation of group-based mindfulness-based stress reduction and cognitive behavioral therapy for the treatment and management of chronic pain disorders: protocol for a systematic review and meta-analysis with indirect comparisons
Source: Syst Rev. 2014 Nov 10;3:134. doi: 10.1186/2046-4053-3-134 (PMC4230908; doi:10.1186/2046-4053-3-134)
Supplement: Additional file 1 — Search strategy. Literature search strategy contains keywords and search strategies used to obtain articles for screening and data extraction for the meta-analysis. [file 2046-4053-3-134-S1.docx]

**Additional file 1**

Mindfulness, CBT & Pain

Search Strategies – Final

2014 Apr 4

Search Name: Mindfulness CBT Pain - Ottawa Pain Clinic

Date Run: 04/04/14 14:34:17.372

Description: Final - 2014 Apr 4

ID Search Hits

#1 [mh Pain] or [mh "Pain Management"] 33311

#2 (pain or painful* or ache or aches or aching):ti,ab,kw 67147

#3 (CNMP or CNCP):ti,ab,kw 9

#4 [mh Fibromyalgia] 629

#5 (fibromyalgia* or fibrosit*):ti,ab,kw 1096

#6 [mh Neuralgia] 760

#7 [mh "Complex Regional Pain Syndromes"] 193

#8 (neuralgia* or neurodynia* or (piriformis next syndrome*) or "pudendal canal entrapment" or "pudendal nerve entrapment" or (pudendal next neuropath*) or sciatica* or causalgia* or "CRPS-I" or "CRPS-1" or CRPSI or CRPS1 or "CRPS-II" or "CRPS-2" or CRPSII or CRPS2 or "CRPS Type I" or "CRPS Type 1" or "CRPS Type II" or "CRPS Type 2"):ti,ab,kw 1587

#9 (algodystroph* or algesidystroph* or algoneurodystroph* or ("cervical sympathetic" next dystroph*) or (Sudek* next atroph*) or ("sympathetic reflex" next dystroph*) or ("reflex sympathetic" next dystroph*) or "RSD syndrome" or "shoulder-hand syndrome" or "hand-shoulder syndrome" or "shoulder-arm syndrome" or "arm-shoulder syndrome"):ti,ab,kw 227

#10 (myalgia* or myodynia*):ti,ab,kw 1319

#11 "chronic compartment" next syndrome*:ti,ab,kw 3

#12 [mh "Polymyalgia Rheumatica"] 47

#13 polymyalgia* near/1 rheumati*:ti,ab,kw 69

#14 [mh "Back Pain"] 2867

#15 (backache* or dorsalgia* or ("failed back" near/3 syndrome*) or lumbago*):ti,ab,kw 954

#16 [mh "Headache Disorders"] 2031

#17 [mh Headache] 1593

#18 (headache* or cephalea* or cephalalgia* or cephalgia* or cephalodynia* or cranialgia* or hemicrania* or (hemi next crania*)):ti,ab,kw 11199

#19 [mh "Migraine Disorders"] 1680

#20 migrain*:ti,ab,kw 2983

#21 [mh "Neck Pain"] 572

#22 (neckache* or cervicalgia* or cervicodynia*):ti,ab,kw 5

#23 [mh "Myofascial Pain Syndromes"] 357

#24 (("myofascial pain" or temporomandibular joint dysfunction or TMJ or Costen's or Costens) next syndrome*):ti,ab,kw 269

#25 [mh Arthralgia] 885

#26 (arthralgia* or polyarthralgia* or (poly next arthralgia*) or arthrodynia*):ti,ab,kw 1460

#27 [mh "Arthritis, Rheumatoid"] 4081

#28 (rheumatism or rheumatoid or rheumarthrit* or (rheum next arthrit*)):ti,ab,kw 6517

#29 ((Caplan* or Felty* or Sjogren* or Sicca) next syndrome*):ti,ab,kw 76

#30 ("adult-onset" near/2 (still* next disease*)):ti,ab,kw 1

#31 {or #1-#30} 86600

#32 [mh Mindfulness] 5

#33 (mindfulness* or Vipassana or "insight meditation"):ti,ab,kw 599

#34 [mh Meditation] 307

#35 meditat*:ti,ab,kw 718

#36 {or #32-#35} 1067

#37 [mh "Cognitive Therapy"] 4916

#38 (cognit* near/3 (therap* or psychotherap* or (psycho next therap*) or treatment*)):ti,ab,kw 9158

#39 CBT:ti,ab,kw 1952

#40 {or #37-#39} 9357

#41 #31 and #36 105

#42 #31 and #40 751

#43 #41 or #42 842

DSR - 46

DARE - 42

CENTRAL – 736

Methods - 2

HTA - 2

NHS EED - 14

Database: Embase Classic+Embase <1947 to 2014 April 03>, Ovid MEDLINE(R) In-Process & Other Non-Indexed Citations and Ovid MEDLINE(R) <1946 to Present>, PsycINFO <1806 to April Week 1 2014> Search Strategy:

--------------------------------------------------------------------------------

1 exp Pain/ or Pain Management/ (1234303)

2 (pain or painful* or ache or aches or aching).tw. (1122735)

3 (CNMP or CNCP).tw. (488)

4 Fibromyalgia/ (20188)

5 (fibromyalgia* or fibrosit*).tw. (19873)

6 exp Neuralgia/ (91375)

7 exp "Complex Regional Pain Syndromes"/ (12306)

8 (neuralgia* or neurodynia* or piriformis syndrome* or pudendal canal entrapment or pudendal nerve entrapment or pudendal neuropath* or sciatica or causalgia* or "CRPS-I" or "CRPS-1" or CRPSI or CRPS1 or "CRPS-II" or "CRPS-2" or CRPSII or CRPS2 or "CRPS Type I" or "CRPS Type 1" or "CRPS Type II" or "CRPS Type 2").tw. (34727)

9 (algodystroph* or algesidystroph* or algoneurodystroph* or cervical sympathetic dystroph* or (Sudek$2 adj atroph*) or sympathetic reflex dystroph* or reflex sympathetic dystroph* or RSD syndrome or shoulder-hand syndrome or hand-shoulder syndrome or shoulder-arm syndrome or arm-shoulder syndrome).tw. (5785)

10 (myalgia* or myodynia*).tw. (15314)

11 chronic compartment syndrome*.tw. (302)

12 Polymyalgia Rheumatica/ (6077)

13 (polymyalgia* adj1 rheumati*).tw. (4848)

14 exp Back Pain/ (100436)

15 (backache* or dorsalgia* or (failed back adj2 syndrome*) or lumbago*).tw. (9726)

16 exp Headache Disorders/ (238379)

17 Headache/ (172038)

18 (headache* or cephalea* or cephalalgia* or cephalgia* or cephalodynia* or cranialgia* or hemicrania* or hemi-crania*).tw. (163672)

19 exp Migraine Disorders/ (65883)

20 migrain*.tw. (69744)

21 Neck Pain/ (17038)

22 (neckache* or cervicalgia* or cervicodynia*).tw. (272)

23 exp Myofascial Pain Syndromes/ (12424)

24 ((myofascial pain or temporomandibular joint dysfunction or TMJ or Costen's or Costens) adj syndrome*).tw. (1802)

25 exp Arthralgia/ (44575)

26 (arthralgia* or polyarthralgia* or poly-arthralgia* or arthrodynia*).tw. (15942)

27 exp Arthritis, Rheumatoid/ (250742)

28 (rheumatism or rheumatoid or rheumarthrit* or rheum arthrit*).tw. (234641)

29 ((Caplan* or Felty* or Sjogren* or Sicca) adj syndrome*).tw. (27636)

30 ("adult-onset" adj1 (still$1 adj disease*)).tw. (1854)

31 or/1-30 (2130896)

32 Mindfulness/ (3418)

33 (mindfulness* or Vipassana or "insight meditation").tw. (8791)

34 Meditation/ (8179)

35 meditat*.tw. (12842)

36 or/32-35 (21028)

37 Cognitive Therapy/ (59619)

38 (cognit* adj2 (therap* or psychotherap* or psycho-therap* or treatment*)).tw. (59905)

39 CBT.tw. (19615)

40 or/37-39 (93111)

41 31 and 36 (2029)

42 31 and 40 (9193)

43 41 or 42 (10868)

44 exp Animals/ not (exp Animals/ and Humans/) (8917918)

45 43 not 44 (10789)

46 (comment or editorial or interview or letter or news).pt. (2773437)

47 45 not 46 (10384)

48 47 use prmz (2227)

49 remove duplicates from 48 (2177) [MEDLINE RECORDS]

50 exp pain/ (1202902)

51 (pain or painful* or ache or aches or aching).tw. (1122735)

52 (CNMP or CNCP).tw. (488)

53 fibromyalgia/ (20188)

54 (fibromyalgia* or fibrosit*).tw. (19873)

55 exp neuralgia/ (91375)

56 (neuralgia* or neurodynia* or piriformis syndrome* or pudendal canal entrapment or pudendal nerve entrapment or pudendal neuropath* or sciatica or causalgia* or "CRPS-I" or "CRPS-1" or CRPSI or CRPS1 or "CRPS-II" or "CRPS-2" or CRPSII or CRPS2 or "CRPS Type I" or "CRPS Type 1" or "CRPS Type II" or "CRPS Type 2").tw. (34727)

57 (algodystroph* or algesidystroph* or algoneurodystroph* or cervical sympathetic dystroph* or (Sudek$2 adj atroph*) or sympathetic reflex dystroph* or reflex sympathetic dystroph* or RSD syndrome or shoulder-hand syndrome or hand-shoulder syndrome or shoulder-arm syndrome or arm-shoulder syndrome).tw. (5785)

58 chronic compartment syndrome*.tw. (302)

59 (polymyalgia* adj1 rheumati*).tw. (4848)

60 (backache* or dorsalgia* or (failed back adj2 syndrome*) or lumbago*).tw. (9726)

61 (headache* or cephalea* or cephalalgia* or cephalgia* or cephalodynia* or cranialgia* or hemicrania* or hemi-crania*).tw. (163672)

62 migrain*.tw. (69744)

63 (neckache* or cervicalgia* or cervicodynia*).tw. (272)

64 ((myofascial pain or temporomandibular joint dysfunction or TMJ or Costen's or Costens) adj syndrome*).tw. (1802)

65 arthralgia/ (41833)

66 (arthralgia* or polyarthralgia* or poly-arthralgia* or arthrodynia*).tw. (15942)

67 rheumatoid arthritis/ or adult onset still disease/ or felty syndrome/ (230774)

68 (rheumatism or rheumatoid or rheumarthrit* or rheum arthrit*).tw. (234641)

69 ((Caplan* or Felty* or Sjogren* or Sicca) adj syndrome*).tw. (27636)

70 ("adult-onset" adj1 (still$1 adj disease*)).tw. (1854)

71 or/50-70 (2095805)

72 Mindfulness/ (3418)

73 (mindfulness* or Vipassana or "insight meditation").tw. (8791)

74 meditation/ (8179)

75 meditat*.tw. (12842)

76 or/72-75 (21028)

77 exp cognitive therapy/ (59763)

78 (cognit* adj2 (therap* or psychotherap* or psycho-therap* or treatment*)).tw. (59905)

79 CBT.tw. (19615)

80 or/77-79 (93242)

81 71 and 76 (2017)

82 71 and 80 (9195)

83 81 or 82 (10848)

84 83 use emczd (6422)

85 limit 84 to yr="2010-current" (2668)

86 84 not 85 (3754)

87 remove duplicates from 85 (2619)

88 remove duplicates from 86 (3714)

89 87 or 88 (6333)

90 exp animals/ or exp animal experimentation/ or exp models animal/ or exp animal experiment/ or nonhuman/ or exp vertebrate/ (38115420)

91 exp humans/ or exp human experimentation/ or exp human experiment/ (28128810)

92 90 not 91 (9988224)

93 89 not 92 (6291)

94 (editorial or letter).pt. (2468212)

95 93 not 94 (5984) [EMBASE RECORDS]

96 exp pain/ or pain management/ (1234303)

97 (pain or painful* or ache or aches or aching).tw. (1122735)

98 (CNMP or CNCP).tw. (488)

99 (fibromyalgia* or fibrosit*).tw. (19873)

100 exp neuralgia/ (91375)

101 "complex regional pain syndrome (type i)"/ (5478)

102 (neuralgia* or neurodynia* or piriformis syndrome* or pudendal canal entrapment or pudendal nerve entrapment or pudendal neuropath* or sciatica or causalgia* or "CRPS-I" or "CRPS-1" or CRPSI or CRPS1 or "CRPS-II" or "CRPS-2" or CRPSII or CRPS2 or "CRPS Type I" or "CRPS Type 1" or "CRPS Type II" or "CRPS Type 2").tw. (34727)

103 (algodystroph* or algesidystroph* or algoneurodystroph* or cervical sympathetic dystroph* or (Sudek$2 adj atroph*) or sympathetic reflex dystroph* or reflex sympathetic dystroph* or RSD syndrome or shoulder-hand syndrome or hand-shoulder syndrome or shoulder-arm syndrome or arm-shoulder syndrome).tw. (5785)

104 (myalgia* or myodynia*).tw. (15314)

105 chronic compartment syndrome*.tw. (302)

106 (polymyalgia* adj1 rheumati*).tw. (4848)

107 (backache* or dorsalgia* or (failed back adj2 syndrome*) or lumbago*).tw. (9726)

108 (headache* or cephalea* or cephalalgia* or cephalgia* or cephalodynia* or cranialgia* or hemicrania* or hemi-crania*).tw. (163672)

109 migrain*.tw. (69744)

110 (neckache* or cervicalgia* or cervicodynia*).tw. (272)

111 ((myofascial pain or temporomandibular joint dysfunction or TMJ or Costen's or Costens) adj syndrome*).tw. (1802)

112 (arthralgia* or polyarthralgia* or poly-arthralgia* or arthrodynia*).tw. (15942)

113 rheumatoid arthritis/ (228150)

114 (rheumatism or rheumatoid or rheumarthrit* or rheum arthrit*).tw. (234641)

115 ((Caplan* or Felty* or Sjogren* or Sicca) adj syndrome*).tw. (27636)

116 ("adult-onset" adj1 (still$1 adj disease*)).tw. (1854)

117 or/96-116 (2110497)

118 mindfulness/ (3418)

119 (mindfulness* or Vipassana or "insight meditation").tw. (8791)

120 meditation/ (8179)

121 meditat*.tw. (12842)

122 or/118-121 (21028)

123 exp cognitive behavior therapy/ (59556)

124 (cognit* adj2 (therap* or psychotherap* or psycho-therap* or treatment*)).tw. (59905)

125 CBT.tw. (19615)

126 or/123-125 (92508)

127 117 and 122 (2024)

128 117 and 126 (9134)

129 127 or 128 (10774)

130 exp Animals/ not (exp Animals/ and Humans/) (8917918)

131 129 not 130 (10695)

132 131 use prmz (2310)

133 131 use emczd (6387)

134 131 not (132 or 133) (1998)

135 remove duplicates from 134 (1996) [PSYCINFO RECORDS]

136 49 or 95 or 135 (10157)

137 limit 136 to yr="2008-current" (5488)

138 136 not 137 (4669)

139 remove duplicates from 137 (4222)

140 remove duplicates from 138 (3442)

141 139 or 140 (7664)

142 141 use prmz (2165) [MEDLINE UNIQUE HITS]

143 141 use emczd (4273) [EMBASE UNIQUE HITS]

144 141 not (142 or 143) (1226) [PSYCINFO UNIQUE HITS]

***************************
